# Signature of Alzheimer’s Disease in Intestinal Microbiome: Results From the AlzBiom Study

**DOI:** 10.3389/fnins.2022.792996

**Published:** 2022-04-19

**Authors:** Christoph Laske, Stephan Müller, Oliver Preische, Victoria Ruschil, Matthias H. J. Munk, Iris Honold, Silke Peter, Ulrich Schoppmeier, Matthias Willmann

**Affiliations:** ^1^Section for Dementia Research, Department of Psychiatry and Psychotherapy, Hertie Institute for Clinical Brain Research, University of Tübingen, Tübingen, Germany; ^2^Department of Psychiatry and Psychotherapy, University of Tübingen, Tübingen, Germany; ^3^German Center for Neurodegenerative Diseases (DZNE), Tübingen, Germany; ^4^Department of Neurology, Epileptology, Hertie Institute for Clinical Brain Research, University of Tübingen, Tübingen, Germany; ^5^Department of Biology, Technische Universität Darmstadt, Darmstadt, Germany; ^6^Institute of Medical Microbiology and Hygiene, University of Tübingen, Tübingen, Germany; ^7^Eurofins Medical Lab Gelsenkirchen, Gelsenkirchen, Germany

**Keywords:** Alzheimer’s disease, intestinal microbiome, taxonomic data, functional data, ensemble learning

## Abstract

**Background:**

Changes in intestinal microbiome composition have been described in animal models of Alzheimer’s disease (AD) and AD patients. Here we investigated how well taxonomic and functional intestinal microbiome data and their combination with clinical data can be used to discriminate between amyloid-positive AD patients and cognitively healthy elderly controls.

**Methods:**

In the present study we investigated intestinal microbiome in 75 amyloid-positive AD patients and 100 cognitively healthy controls participating in the AlzBiom study. We randomly split the data into a training and a validation set. Intestinal microbiome was measured using shotgun metagenomics. Receiver operating characteristic (ROC) curve analysis was performed to examine the discriminatory ability of intestinal microbiome among diagnostic groups.

**Results:**

The best model for discrimination of amyloid-positive AD patients from healthy controls with taxonomic data was obtained analyzing 18 genera features, and yielded an area under the receiver operating characteristic curve (AUROC) of 0.76 in the training set and 0.61 in the validation set. The best models with functional data were obtained analyzing 17 GO (Gene Ontology) features with an AUROC of 0.81 in the training set and 0.75 in the validation set and 26 KO [Kyoto Encyclopedia of Genes and Genomes (KEGG) ortholog] features with an AUROC of 0.83 and 0.77, respectively. Using ensemble learning for these three models including a clinical model with the 4 parameters age, gender, BMI and ApoE yielded an AUROC of 0.92 in the training set and 0.80 in the validation set.

**Discussion:**

In conclusion, we identified a specific Alzheimer signature in intestinal microbiome that can be used to discriminate amyloid-positive AD patients from healthy controls. The diagnostic accuracy increases from taxonomic to functional data and is even better when combining taxonomic, functional and clinical models. Intestinal microbiome represents an innovative diagnostic supplement and a promising area for developing novel interventions against AD.

## Introduction

Alzheimer’s disease (AD) is the leading cause of dementia in the elderly. Neuropathological hallmarks of AD are the deposition of extracellular β amyloid (Aβ) plaques, intracellular accumulation of tau-containing neurofibrillary tangles (NFTs), cortical atrophy and neuroinflammation in the brain (Querfurth and Laferla, 2010). The mechanisms leading to this AD pathology are still unknown. A multifactorial genesis seems likely, with inflammatory changes in the brain including altered microglia function playing an important role (McGeer and McGeer, 1995; Wyss-Coray, 2006). The mechanisms triggering these inflammatory changes are also unknown.

The intestine with its mucosal lymphoid tissue is one of the most important components of the immune system, containing about 70–80% of the body’s immune system (Sochocka et al., 2019). The colon contains about 39 × 1,012 bacteria and many more microorganisms (Sender et al., 2016). The totality of these microorganisms is called intestinal microbiome and contains about 100 × more genetic information than the human genome. The gut microbiome plays a pivotal role in supporting human health by regulation of metabolic, endocrine, immune and neurotrophic functions, e.g., by production of short-chain fatty acids (SCFAs) (Silva et al., 2020) and brain-derived neurotrophic factor (BDNF) (Lee et al., 2018; Ranuh et al., 2019). The influence of the gut microbiome is not restricted to the gastrointestinal (GI) tract, it also plays an important role in the bidirectional communication between the GI tract and the brain (microbiota-gut-brain axis; Morais et al., 2021).

Taxonomic and functional changes to the composition of the gut microbiome have been associated with multiple human diseases, e.g., with chronic inflammatory diseases such as Crohn’s disease, ulcerative colitis, and rheumatoid arthritis (Manichanh et al., 2006; Vigsnæs et al., 2012; Zhang et al., 2015). The composition of the gut microbiome can be impacted by host and environmental factors. It also holds different functional features across various life periods (Kundu et al., 2017). While gut microbiome remains mostly stable during the adulthood, it starts at the age of 65 years—interestingly the predilection age for AD—to shift into a less diverse and resilient state, which makes the microbiome more susceptible to environmental factors (Thursby and Juge, 2017).

Changes in the gut microbiome seem to be also linked with AD. Several recent studies have demonstrated changes in intestinal microbiome composition in animal models of AD (Harach et al., 2017; Chen et al., 2020), in human AD patients (Vogt et al., 2017; Li et al., 2019; Liu et al., 2019; Ling et al., 2020) and in MCI patients (Li et al., 2019; Liu et al., 2019). Most of these studies examined taxonomic data, only a few also functional data and none investigated the combination of taxonomic, functional and clinical data using an ensemble learning approach. Composition of intestinal microbiome is altered in animal models of AD even before the presence of amyloid plaques in the brain and thus seems to be an early phenomenon within AD pathogenesis (Chen et al., 2020). In addition, it has been shown that animal models of AD develop in the absence of intestinal microbiome less amyloid pathology in the brain than animal models of AD with existing or replaced intestinal microbiome (Harach et al., 2017; Dodiya et al., 2019, 2020).

The aim of the present study was to examine how well taxonomic and functional intestinal microbiome data and their combination with clinical data can be used to discriminate between amyloid-positive AD patients and cognitively healthy elderly controls.

## Materials and Methods

### Participants

Seventy-five amyloid-positive AD patients and 100 cognitively healthy controls were included in the study (Table 1). These participants were recruited from the AlzBiom study. AlzBiom is an observational longitudinal study performed in the Section for Dementia Research at the Department of Psychiatry and Psychotherapy in Tübingen. The aim of this ongoing study is to enroll mild cognitive impairment (MCI) patients, AD dementia patients and control subjects without subjective or objective cognitive decline. All participants underwent Mini-Mental State Examination (MMSE)-scoring (Folstein et al., 1975) and clinical assessment of cognitive status by means of the Clinical Dementia Rating (CDR) scale (Morris, 1993, 1997). For further statistical analysis, we randomly split the data into training set consisting of 132 individuals (AD: *n* = 59, HC: *n* = 73) and validation set comprising 43 individuals (AD: *n* = 16, HC: *n* = 27).

**TABLE 1:** Clinical and demographic characteristics of healthy control (HC) individuals and amyloid-positive Alzheimer’s disease (AD) patients.

	HC	Amyloid-positive AD patients	*p*-value
N	100	75	
Age, years, mean (SD)	71.0 (4.4)	68.6 (8.6)	0.0323
Gender (m/f)	46/54	41/34	0.2565
MMSE, mean (SD)	28.9 (1.9)	22.6 (5.1)	<0.0001
GDS, mean (SD)	2.1 (2.6)	2.8 (2.1)	0.0563
Body mass index (BMI), mean (SD)	25.9 (4.5)	24.9 (4.3)	0.1547
ApoE (e4/e4 carriers/single e4 carriers/non-e4-carriers; n)	2/18/38	5/30/18	0.0036
Arterial hypertension (yes/no)	42/58	29/46	0.6567
Diabetes mellitus (yes/no)	5/95	1/74	0.1871
Rheumatoid arthritis (yes/no)	7/93	2/73	0.6314
NSAIDs (yes/no)	24/76	18/57	1.000
Anticoagulants (yes/no)	4/96	2/73	0.6314
Antihypertensives (yes/no)	41/59	33/42	0.6910
Antidiabetics (yes/no)	4/96	0/75	0.0797
Statins (yes/no)	11/89	12/63	0.3326
Antidepressants (yes/no)	6/94	23/52	<0.0001
AChE inhibitors (yes/no)	0/100	43/32	<0.0001
CSF Aβ-42 (pg/ml)	–	478.7 (101.7)	–
CSF h-Tau (pg/ml)	–	727.1 (386.5)	–
CSF p-Tau (pg/ml)	–	90.9 (38.9)	–

*Values are expressed as mean (standard deviation). N, number; HC, healthy control individuals; AD, Alzheimer’s disease patients; m/f, male/female; MMSE, Mini Mental State Examination; GDS, Geriatric Depression Scale; NSAIDs, Non-steroidal antiphlogistics; AChE, Acetylcholinesterase; CSF, cerebrospinal fluid.*

AD patients fulfilled the NIA-AA core clinical criteria for probable AD dementia (McKhann et al., 2011), had a global CDR score of 0.5–1.0 and had evidence of cerebral Aβ accumulation with Aβ42 cerebrospinal fluid (CSF) levels of <600 pg/ml. HC individuals never reported subjective cognitive decline (SCD), had no history of neurological or psychiatric disease or any sign of cognitive decline and had a global CDR score of 0 and a MMSE score of ≥27.

The regional ethical committee approved the study and written informed consent was obtained from each individual.

### Determination of Apolipoprotein ε4 Genotype

The procedure for determining the apolipoprotein (ApoE) genotype was performed as previously described (Operto et al., 2019). Briefly, total DNA was obtained from the blood cellular fraction by proteinase K digestion followed by alcohol precipitation, using the QIAamp DNA Blood Maxi Kit. APOE genotyping was carried out using Applied Biosystems, Assay-on-demand TaqMan^®^ SNP genotyping Assays, C_3084793_20 and C_904973_10 corresponding to APOE SNPs rs429358 and rs7412, respectively, and run on a StepOne Real-Time PCR Systems instrument. The ApoE ε4 positive genotype was assigned if at least one ε4 allele was present.

### Cerebrospinal Fluid Collection and Analysis

CSF was obtained by lumbar puncture with aseptic technique at the L3-L4 or L4-L5 intervertebral spinous process space, using a 22- or 21-gauge needle. The determination of the CSF concentrations of Amyloid-beta 1-42 (Aβ42) (cut-off: <600 pg/ml), total tau (t-tau) and phospho-tau181 (p-tau) was performed by commercial ELISAs (INNOTEST^§^ β-AMYLOID[1-42], Fujirebio Germany, detection limit 225 pg/ml; INNOTEST^§^ hTAU Ag, Fujirebio Germany, detection limit 40 pg/ml; INNOTEST^§^ PHOSPHO-TAU[181P], Fujirebio Germany, detection limit 15 pg/ml) according to the manufacturer’s instructions.

### Stool Collection, DNA Extraction, and Shotgun Metagenomic Sequencing

We collected stool samples in a sterile plastic device (Commode Specimen Collection System, Thermo Fisher Scientific, Pittsburgh, United States). The majority of stool samples were collected at the participants’ home using the DNA/RNA Shield Fecal Collection Tube R1101 (Zymo Research, Irvine, United States) and immediately sent to our laboratory by post. Samples were stored at −20°C and DNA was extracted on the same day using ZymoBiomics DNA Miniprep Kit D4300 (Zymo Research, Irvine, United States). DNA extraction was carried out at the end of sample collection in batches of 12–18 samples. Shotgun metagenomic sequencing was carried out in one batch at the GATC Biotech AG (Konstanz, Germany) using the NEBNext Ultra DNA Library kit (New England Biolabs, Ipswich, United States) for DNA library preparation and an Illumina HiSeq platform for sequencing. A paired-end sequencing approach with a targeted read length of 150 bp and an insert size of 550 bp was conducted. We aimed for a median sequencing depth of 40–50 million reads per sample.

### Metagenomic Assembly

Trimmomatic (version 0.35) was used to acquire high-quality reads through adapter removing and through a sliding window trimming (Bolger et al., 2014). Reads were trimmed to a minimum length of 100 bp. Quality control of trimmed reads was performed with FastQC version 0.11.5.^1^ We used SPAdes (version 3.9.0) to assemble metagenomic scaffolds with a minimum length of 1,000 bp (Bankevich et al., 2012).

### Taxonomic Classification

Human contamination was removed by mapping reads against the human genome (GRCh38) using KneadData.^2^ Taxonomic profiling was carried out with Kaiju (version 1.5.0) using the greedy mode with a minimum alignment length of 11 amino acids, a maximum of 1 mismatch, and a match score of 65 (Menzel et al., 2016). The NCBI RefSeq was used as reference database. Counts for taxonomic units were normalized to a relative abundance through dividing the hits by the sample read count and multiplying the quotient by 10^6^. The resulting unit is hits per million reads (HPM).

### Functional Classification

Community functional profiles were analyzed using HUMAnN 2.0 (version 0.11.2) using the standard parameters (Franzosa et al., 2018). According to OUT and Phylogenetic Investigation of Communities by Reconstruction of Unobserved States (PICRUSt) (Langille et al., 2013), we identified the functional categories based on a comparison of the Kyoto Encyclopedia of Genes and Genomes (KEGG) ortholog (KO)^3^ and of Gene Ontology (GO) Resource.^4^

### Statistical Analysis

For demographic characteristics and clinical information, the statistical software package SPSS (version 23) was used for data analyses. For all tests, the level of statistical significance was set to *p* < 0.05. Levene’s test served to assess homogeneity of variances. In case of continuous variables (i.e., age and BMI) *t*-tests for independent samples were used to detect differences between both groups. The non-parametric Mann-Whitney-*U*-test was conducted to detect group differences in MMSE and geriatric depression scale (GDS). The Pearson chi-square test was used to detect group differences in gender distribution, ApoE status and medication. To investigate the potential influence of antidepressant use and AChE inhibitor use on the relative abundance of genera, we performed a Hotelling-T^2^ test on the first 101 principal components (to capture at least 95% of the variance) of 1,014 normalized and isometric-logratio transformed genera data.

As features we investigated taxonomic data (phyla, genera and species), functional data (EGGNOG, GO, KO) and clinical meta data (age, gender, BMI, ApoE). We randomly split the data into a training and a validation set (ratio was 3:1) using caret library (createDataPartition). Feature pre-selection was performed in the training set using Wilcoxon test, and balances were calculated as part of compositional data analysis (Rivera-Pinto et al., 2018). Resulting models were then trained applying a logistic regression approach. In this training phase further feature selection was achieved. For this we performed fivefold cross-validation with 30 repeats of models shrinking the number of features step by step. Best models (Genera, GO, KO and clinical meta data) were then assessed by application to the validation data sets.

In the last step we joined the best performing models in an ensemble learning model, as described in http://www.scholarpedia.org/article/Ensemble_learning. Like with individual models, we trained the ensemble model with training data and assessed the quality using the validation data.

Receiver operating characteristic (ROC) curve analysis was performed to examine the discriminatory ability of intestinal microbiome among diagnostic groups. From the best models (Genera, GO, KO), bar plots were generated and heat maps showed their correlation with clinical and biomarker data. The pre-processing and statistical analysis of data was done using customized R scripts and HeidiSQL (1.3) as a database management tool in connection with RMariaDB (1.1.1). The model training and its feature selection was coded in R scripts relying on mlr (2.18.0) package. ROC curves were calculated employing OptimalCutpoints (1.1-4) and plotted with ggplot2 (3.3.5). Codes and data as well as some instructions are available at https://github.com/UliSchopp/AlzBiom. We give a full account of the method and all steps involved in Supplementary Material. The datasets presented in this study can be found at https://www.ebi.ac.uk/ena, PRJEB47976.

## Results

Characteristics of the study sample are presented in Table 1. HCs revealed significantly higher age (*p* = 0.032) and MMSE scores (*p* < 0.0001) compared to amyloid-positive AD patients. Gender was equally distributed between the investigated groups (*p* = 0.257). As expected, amyloid-positive AD patients took more frequently AChE inhibitors (*p* < 0.0001) and antidepressants (*p* < 0.0001). However, we found no statistically significant influence of antidepressant use (*p* = 0.25) and AChE inhibitor use (*p* = 0.21) on the relative abundance of genera.

**FIGURE 1:**
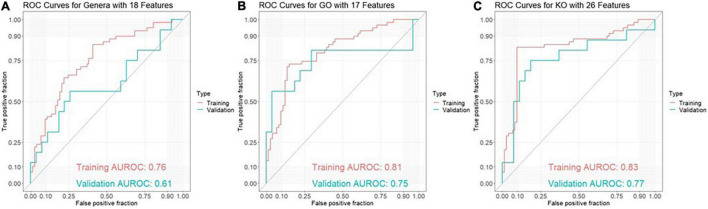
ROC curves for discrimination between amyloid-positive Alzheimer’s disease (AD) patients and healthy controls in a training set (AD: *n* = 59, HC: *n* = 73) and an independent validation set (AD: *n* = 16, HC: *n* = 27) based on **(A)** 18 genera features, **(B)** 17 GO (Gene Ontology) features and **(C)** 26 KO [Kyoto Encyclopedia of Genes and Genomes (KEGG) ortholog] features.

**FIGURE 2:**
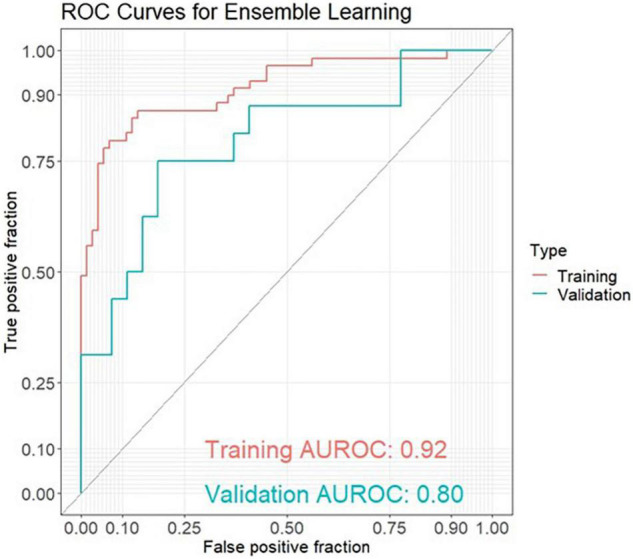
ROC curves for ensemble learning for discrimination between amyloid-positive Alzheimer’s disease (AD) patients and healthy controls in a training set (AD: *n* = 59, HC: *n* = 73) and an independent validation set (AD: *n* = 16, HC: *n* = 27) including 4 models with 18 genera features, 17 GO (Gene Ontology) features, 26 KO [Kyoto Encyclopedia of Genes and Genomes (KEGG) ortholog] features and 4 clinical features (age, gender, BMI, ApoE).

The best model for discrimination of amyloid-positive AD patients from healthy controls with taxonomic data was obtained analyzing 18 genera features, and yielded an area under the receiver operating characteristic curve (AUROC) of 0.76 (95% CI: 0.67–0.84) in the training set and 0.61 (95% CI: 0.42–0.80) in the validation set (Figure 1A). The best models with functional data were obtained analyzing 17 GO features with an AUROC of 0.81 (95% CI: 0.73–0.88) in the training set and 0.75 (95% CI: 0.56–0.94) in the validation set (Figure 1B) and 26 KO features with an AUROC of 0.83 (95% CI: 0.74–0.91) and 0.77 (95% CI: 0.60–0.93), respectively (Figure 1C). Using ensemble learning for these three models including a clinical model with the 4 parameters age, gender, BMI and ApoE yielded an AUROC of 0.92 (95% CI: 0.87–0.97) in the training set and 0.80 (95% CI: 0.65–0.94) in the validation set (Figure 2).

**FIGURE 3:**
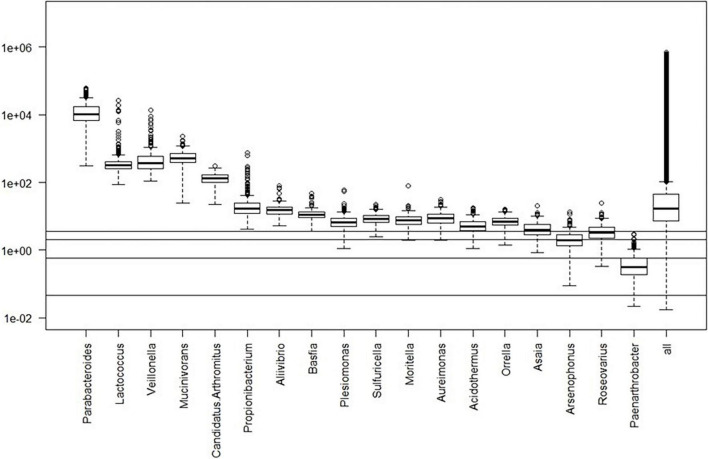
Box plots of the absolute abundances of the 18 genera used in the genera model compared to all genera in our study cohort. The 0.1, 1.0, 5.0, and 10.0% quantiles are given as horizontal lines starting from below.

Box plots of the absolute abundances of the 18 genera used in the genera model compared to all genera in our study cohort are shown in Figure 3. It turns out that all model genera are quite evenly distributed around the median of all genera. The differences of logarithmised medians of the features included in the genera, GO and KO model are shown in Tables 2–4. The non-significant associations between genera, GO and KO features and AD clinical and biomarker characteristics are presented in Tables 5–7.

**TABLE 2:** The differences of logarithmised medians of the features included in the genera model.

Genus	Phylum	Differences of logarithmised medians
Aliivibrio	Proteobacteria	–0.0816
Propionibacterium	Actinobacteria	–0.0496
Orrella	Proteobacteria	–0.0444
Veillonella	Firmicutes	–0.0416
Mucinivorans	Bacteroidetes	–0.0413
Paenarthrobacter	Actinobacteria	–0.0265
Plesiomonas	Proteobacteria	–0.0224
Roseovarius	Proteobacteria	–0.018
Lactococcus	Firmicutes	–0.0165
Sulfuricella	Proteobacteria	–0.0045
Moritella	Proteobacteria	0.0096
Parabacteroides	Bacteroidetes	0.0195
Basfia	Proteobacteria	0.021
Arsenophonus	Proteobacteria	0.0224
Acidothermus	Actinobacteria	0.0282
Aureimonas	Proteobacteria	0.0508
Candidatus Arthromitus	Firmicutes	0.0653
Asaia	Proteobacteria	0.0853

*Negative values indicate higher levels in healthy controls; positive values indicate higher levels in Alzheimer’s disease (AD) patients.*

**TABLE 3:** The differences of logarithmised medians of the features included in the GO (Gene Ontology) model.

GO label	Function	Differences of logarithmised medians
GO 0047980	Hippurate hydrolase activity	–0.1968
GO 0031647	Regulation of protein stability	–0.1494
GO 0030412	Formimidoyltetrahydrofolate cyclodeaminase activity	–0.1463
GO 0008953	Penicillin amidase activity	–0.1402
GO 0008514	Organic anion transmembrane transporter activity	–0.1301
GO 0004793	Threonine aldolase activity	–0.1083
GO 0035556	Intracellular signal transduction	–0.1056
GO 0070008	Serine type exopeptidase activity	–0.0876
GO 0004038	Allantoinase activity	–0.0793
GO 0071973	Bacterial type flagellum dependent cell motility	–0.0539
GO 0008452	RNA ligase activity	0.0249
GO 0004866	Endopeptidase inhibitor activity	0.0562
GO 0004008	Copper exporting ATPase activity	0.0600
GO 0050385	Ureidoglycolate lyase activity	0.0683
GO 0008792	Arginine decarboxylase activity	0.0961
GO 0030596	Alpha-L-rhamnosidase activity	0.1039
GO 0033727	Aldehyde dehydrogenase FAD independent activity	0.1982

*Negative values indicate higher levels in healthy controls; positive values indicate higher levels in Alzheimer’s disease (AD) patients.*

**TABLE 4:** The differences of logarithmised medians of the features included in the KO [Kyoto Encyclopedia of Genes and Genomes (KEGG) ortholog] model.

KO label	Function	Differences of logarithmised medians
K02573	Ferredoxin type protein NapG	–0.2774
K01838	Beta phosphoglucomutase	–0.2398
K02773	PTS system_galactitol specific IIA component	–0.2318
K07497	Putative transposase	–0.2010
K01089	Imidazoleglycerol phosphate dehydratase histidinol phosphatase	–0.1793
K07448	Restriction system protein	–0.1769
K06223	DNA adenine methylase	–0.1179
K01051	Pectinesterase	–0.1030
K02041	Phosphonate transport system ATP binding protein	–0.0841
K11065	Thiol peroxidase_atypical 2 Cys peroxiredoxin	–0.0733
K07243	High affinity iron transporter	–0.0676
K00244	Fumarate reductase flavoprotein subunit	–0.0548
K01673	Carbonic anhydrase	–0.0487
K00384	Thioredoxin reductase NADPH	0.0208
K01649	2 isopropylmalate synthase	0.0219
K02563	UDP N acetylglucosamine N acetylmuramyl pentapeptide pyrophosphoryl undecaprenol N acetylglucosamine transferase	0.0235
K02120	V A type H Na transporting ATPase subunit D	0.0322
K02909	Large subunit ribosomal protein L31	0.0340
K00031	Isocitrate dehydrogenase	0.0410
K03296	Hydrophobic amphiphilic exporter 1 mainly G bacteria_HAE1 family	0.0474
K18369	Alcohol dehydrogenase	0.0634
K08963	Methylthioribose 1 phosphate isomerase	0.0675
K13940	Dihydroneopterin aldolase 2 amino 4 hydroxy 6 hydroxymethyldihydropteridine diphosphokinase	0.1349
K00549	5 methyltetrahydropteroyltriglutamate homocysteine methyltransferase	0.1676
K02083	Allantoate deiminase	0.2029
K10547	Putative multiple sugar transport system permease protein	0.2212

*Negative values indicate higher levels in healthy controls; positive values indicate higher levels in Alzheimer’s disease (AD) patients.*

**TABLE 5:** The associations between genera and Alzheimer’s disease (AD) clinical and biomarker characteristics.

Genus	Age	MMSE	ApoE4	CSF Aβ	CSF t-tau	CSF p-tau
Acidothermus	–0.025	–0.164	0.000	0.233	–0.151	0.009
Aliivibrio	0.097	0.026	–0.037	0.026	–0.020	0.013
Arsenophonus	–0.046	0.001	0.111	0.110	–0.206	–0.154
Asaia	0.093	–0.058	0.013	0.027	–0.198	–0.113
Aureimonas	–0.116	–0.093	0.061	–0.010	–0.306	–0.075
Basfia	0.029	–0.063	–0.005	0.032	–0.120	–0.014
Candidatus Arthromitus	0.124	–0.026	0.039	0.055	–0.243	–0.108
Lactococcus	–0.057	0.086	–0.044	0.007	–0.177	–0.020
Moritella	0.000	–0.074	0.059	0.242	–0.213	–0.237
Mucinivorans	–0.050	–0.073	0.003	0.239	–0.428	–0.163
Orrella	–0.107	0.076	0.005	0.088	–0.174	0.035
Paenarthrobacter	–0.029	0.003	–0.05	0.158	–0.056	0.078
Parabacteroides	0.022	–0.121	0.022	0.116	–0.306	–0.160
Plesiomonas	0.032	0.058	0.058	–0.003	–0.236	–0.126
Propionibacterium	–0.163	0.000	0.196	0.133	–0.180	–0.154
Roseovarius	0.009	0.019	–0.056	0.057	–0.260	–0.062
Sulfuricella	0.036	0.046	0.021	–0.025	–0.170	–0.009
Veillonella	0.002	–0.011	–0.091	0.015	–0.173	–0.125

**TABLE 6:** The associations between GO (Gene Ontology) features and Alzheimer’s disease (AD) clinical and biomarker characteristics.

GO label	Function	Age	MMSE	ApoE4	CSF Aβ	CSF t-tau	CSF p-tau
GO 0070008	Serine type exopeptidase activity	–0.123	0.163	–0.091	0.082	–0.152	0.032
GO 0030412	Formimidoyltetrahydrofolate cyclodeaminase activity	–0.002	0.230	0.032	–0.137	0.048	–0.194
GO 0033727	Aldehyde dehydrogenase FAD independent activity	0.043	0.031	0.082	–0.107	0.037	0.154
GO 0004793	Threonine aldolase activity	–0.047	0.279	–0.073	–0.093	0.059	–0.098
GO 0008514	Organic anion transmembrane transporter activity	–0.068	0.083	–0.126	–0.043	0.049	0.099
GO 0035556	Intracellular signal transduction	0.010	0.234	–0.093	0.169	–0.154	–0.249
GO 0008953	Penicillin amidase activity	0.010	–0.098	0.029	–0.003	0.135	0.057
GO 0004866	Endopeptidase inhibitor activity	–0.189	–0.222	–0.130	0.326	0.064	0.159
GO 0030596	Alpha L rhamnosidase activity	–0.064	–0.125	0.191	–0.151	0.227	–0.025
GO 0047980	Hippurate hydrolase activity	0.059	0.210	–0.148	0.157	–0.054	–0.060
GO 0008792	Arginine decarboxylase activity	–0.044	–0.119	–0.051	0.291	–0.306	–0.229
GO 0004038	Allantoinase activity	–0.024	0.137	–0.103	–0.035	0.261	0.222
GO 0050385	Ureidoglycolate lyase activity	0.013	–0.008	–0.005	–0.218	–0.122	–0.233
GO 0008452	RNA ligase activity	0.018	–0.118	–0.035	–0.031	–0.032	–0.057
GO 0031647	Regulation of protein stability	–0.016	0.118	0.002	–0.083	0.161	0.308
GO 0071973	Bacterial type flagellum dependent cell motility	0.026	0.173	0.003	0.020	–0.066	–0.022
GO 0004008	Copper exporting ATPase activity	–0.116	0.002	0.263	–0.070	–0.223	–0.007

**TABLE 7:** The associations between KO [Kyoto Encyclopedia of Genes and Genomes (KEGG) ortholog] features and Alzheimer’s disease (AD) clinical and biomarker characteristics.

KO label	Function	Age	MMSE	ApoE4	CSF Aβ	CSF t-tau	CSF p-tau
K07497	Putative transposase	0.035	0.126	–0.147	0.048	0.331	–0.149
K10547	Putative multiple sugar transport system permease protein	0.037	–0.093	0.143	–0.144	–0.053	–0.256
K02773	PTS system_ galactitol specific IIA component	0.061	0.202	–0.102	0.067	0.135	0.125
K02909	Large subunit ribosomal protein L31	0.035	–0.074	0.024	–0.126	–0.069	–0.175
K02083	Allantoate deiminase	0.022	0.001	0.047	–0.238	0.038	–0.238
K02573	Ferredoxin type protein NapG	0.022	0.165	–0.105	0.132	–0.085	0.057
K01838	Beta phosphoglucomutase	0.020	0.195	–0.019	–0.083	0.267	–0.145
K02120	V A type H Na transporting ATPase subunit D	0.179	–0.144	–0.042	–0.084	0.093	0.114
K00384	Thioredoxin reductase NADPH	0.019	–0.291	0.012	–0.060	–0.103	0.073
K13940	Dihydroneopterin aldolase 2 amino 4 hydroxy 6 hydroxymethyldihydropteridine diphosphokinase	–0.003	–0.238	0.108	0.021	0.058	0.237
K07243	High affinity iron transporter	–0.006	0.142	–0.188	0.044	0.200	0.375
K00549	5 methyltetrahydropteroyltri-glutamate homocysteine methyltransferase	–0.042	–0.041	–0.096	0.058	0.221	–0.076
K07448	Restriction system protein	0.144	–0.011	0.075	–0.057	–0.091	–0.043
K01673	Carbonic anhydrase	0.002	0.04	–0.121	0.01	0.197	0.184
K11065	Thiol peroxidase_atypical 2 Cys peroxiredoxin	0.095	0.033	0.091	–0.084	0.064	–0.051
K01051	Pectinesterase	–0.050	0.218	–0.057	0.057	0.084	–0.086
K01649	2 isopropylmalate synthase	0.101	–0.198	0.010	0.218	–0.170	0.070
K02563	UDP N acetylglucosamine N acetylmuramyl pentapeptide pyrophosphoryl undecaprenol N acetylglucosamine transferase	0.097	–0.138	–0.012	–0.099	0.127	0.175
K00244	Fumarate reductase flavoprotein subunit	–0.06	0.248	–0.08	0.16	–0.166	–0.121
K18369	Alcohol dehydrogenase	–0.082	0.001	–0.009	0.083	–0.061	–0.134
K08963	Methylthioribose 1 phosphate isomerase	0.063	–0.176	–0.032	0.031	–0.102	–0.222
K02041	Phosphonate transport system ATP binding protein	–0.074	0.205	–0.105	–0.164	0.304	0.083
K06223	DNA adenine methylase	0.08	0.135	–0.128	0.064	0.022	–0.158
K03296	Hydrophobic amphiphilic exporter 1 mainly G bacteria HAE1 family	0.088	–0.135	–0.06	0.135	–0.135	–0.107
K00031	Isocitrate dehydrogenase	0.01	–0.026	–0.012	–0.008	0.053	–0.009
K01089	Imidazoleglycerol phosphate dehydratase histidinol phosphatase	0.11	–0.02	–0.027	–0.001	0.084	–0.012

## Discussion

This study investigated the capability of taxonomic and functional intestinal microbiome data and their combination with clinical data in discriminating amyloid-positive AD patients from cognitively healthy elderly controls. To our knowledge, this is the first study addressing this question using taxonomic and functional data of shotgun metagenomics in a training cohort and an independent validation cohort. Within taxonomic data, we identified a genera model and within functional data, we identified a GO and KO model showing best results for discrimination of amyloid-positive AD patients from healthy controls. Functional data showed a higher diagnostic accuracy than taxonomic data. In a next step, we combined taxonomic, functional and clinical models with an ensemble learning approach. This ensemble model performed better than any of its parts in the training data and with respect to genera and clinical model also in the validation data. Our finding of an added value of including clinical variables to gut microbiome data is in line with previous studies, demonstrating an improved accuracy for AD diagnosis adding clinical variables to blood-based biomarkers (O’Bryant et al., 2011) or MRI data (Vemuri et al., 2008).

Several recent studies have demonstrated an altered intestinal microbiome composition in animal models of AD (Harach et al., 2017) and in human AD patients (Vogt et al., 2017; Li et al., 2019; Liu et al., 2019; Ling et al., 2020) and MCI patients (Li et al., 2019; Liu et al., 2019). Three studies investigated the diagnostic accuracy for discrimination of clinically diagnosed AD patients from healthy controls using genera data with AUROC values ranging from 0.78 to 0.94 (Li et al., 2019; Liu et al., 2019; Ling et al., 2020). However, all three studies had some limitations: They used limited bacterial 16S rRNA sequencing technology, they did not validate their results in an independent cohort with the same diagnosis and they did not examine the diagnostic accuracy of functional data. Instead, Li and colleagues (Li et al., 2019) showed that their genera model from AD patients was able to predict 28 of 30 patients with MCI, suggesting a similar pattern of gut microbiome changes in AD and MCI patients. In contrast, Liu and colleagues (Liu et al., 2019) could discriminate AD patients and MCI patients using a genera model with an AUROC of 0.93, suggesting a different pattern of gut microbiome changes in AD and MCI patients. Both results are conflicting and also surprising, because MCI is a heterogeneous syndrome with varying clinical outcomes: up to 60% of MCI patients develop dementia within a 10-year period, however, many people remain cognitively stable or regain normal cognitive function (Manly et al., 2008; Mitchell and Shiri-Feshki, 2009; Korolev et al., 2016). We hope in the future to be able to resolve these conflicting results by providing more information for differentiating microbiome patterns of MCI patients and testing how much overlap there is with more advanced pathology.

Interestingly, 10 of the 18 taxa in our genera model belong to the phylum proteobacteria. Of these, 5 show higher levels in healthy individuals and 5 in AD patients. This means, that 50% of taxa with higher levels in healthy controls and 62.5% of taxa with higher levels in AD patients belong to the phylum proteobacteria. However, on the phylum level we found no significant difference of the relative abundance of proteobacteria between AD patients and healthy controls (*p* = 0.41). This result indicates that differences of taxa may play rather a role on the genera level than on phylum level. The potential meaning of proteobacteria in AD has been shown in a previous study, describing increased proteobacteria levels in AD patients, which correlated with the severity of cognitive impairment (Liu et al., 2019). From the 17 features in the GO model, aldehyde dehydrogenase showed the highest activity in AD patients. This is an interesting finding, as this enzyme has been associated with neuroprotective effects due to its aldehyde detoxification and with neurodegenerative diseases such as AD (Michel et al., 2010; Grünblatt and Riederer, 2016). From the 26 features in the KO model, putative multiple sugar transport system permease protein showed the highest activity in AD patients. This protein belongs to the ATP-binding cassette (ABC) transporters which have been associated with AD pathogenesis due to their ability to transport Aβ peptides out of the brain (Aβ brain clearance; Behl et al., 2021).

The intestinal microbiome may have a direct impact on amyloid pathology and neuroinflammation in the brain. This hypothesis is supported by recent preclinical studies showing that the absence or presence of intestinal microbiome influenced the degree of cerebral amyloid pathology in animal models of AD (Harach et al., 2017; Dodiya et al., 2019, 2020). Germ-free Alzheimer’s mice had less amyloid plaques in the brain than Alzheimer’s mice with normal intestinal microbiome. The colonization of germ-free Alzheimer’s mice with microbiota from conventionally reared Alzheimer’s mice resulted in a significant increase in cerebral amyloid load in the brain, but not the microbiota of control mice (Harach et al., 2017). In addition, treatment of male Alzheimer’s mice with an antibiotic cocktail resulted in changes of gut microbiome and was associated with reduced amyloid pathology in the brain and transplants of fecal microbiota from male Alzheimer’s mice restored the gut microbiome and partially restored amyloid pathology in the brain (Dodiya et al., 2019). A recent study showed that when the gut microbiota from AD patients were transplanted into Alzheimer’s mice, the recipient mice showed more severe cognitive impairment, activated intestinal NLRP3 inflammasome, increased levels of inflammatory factors in peripheral blood and activated microglia in the hippocampus, and these effects could be reversed by transplantation of healthy human gut microbiota (Shen et al., 2020).

Which mechanisms could mediate the effects of intestinal microbiome on amyloid pathology in AD patients? A recent study has identified the bacterial products lipopolysaccharide (LPS) and the short chain fatty acids (SCFAs) acetic acid and valeric acid to be positively correlated with amyloid load in the brain of AD patients, whereas butyric acid showed an inverse correlation (Marizzoni et al., 2020). In addition, an *in vitro* study revealed an anti-Aβ aggregation efficacy for the SCFAs valeric acid, butyric acid, and propionic acid (Ho et al., 2018). This anti-amyloidogenic effect of some SCFAs could be explained in part by their influence on maturation and function of microglia in the brain (Erny et al., 2015), which can clean up Aβ fibrils and typically surrounds amyloid plaques in the brains of AD patients (Calsolaro and Edison, 2016). In contrast, LPS is part of the outer membrane of Gram-positive bacteria and has been shown to trigger systemic inflammation and the release of proinflammatory cytokines (Zhao and Lukiw, 2015) and to potentiate amyloid fibrillogenesis (Asti and Gioglio, 2014).

The findings that gut microbiome is changed in AD and directly influences the degree of amyloid pathology and neuroinflammation in the brain indicate that it could represent a promising target for the prevention or treatment of AD. In this context, the use of a probiotic cocktail for 12 weeks has shown beneficial effects on cognitive performance in AD patients (Akbari et al., 2016). The beneficial effect of gut microbiota on cognition could be explained in part by the upregulation of hippocampal BDNF and cAMP-response element-binding protein (CREB), as shown for different *lactobacillus* species (Lee et al., 2018; Ranuh et al., 2019). However, a meta-analysis examining the effects of probiotic supplementation on cognition in dementia could not find a significant beneficial effect (Krüger et al., 2021).

Despite its significant implications, the present study has some limitations to be discussed. First, this study used a cross-sectional design. To determine whether the characteristic intestinal microbiome in AD is the cause or result of the disease, well designed longitudinal studies with adequate sample size are needed. Second, CSF was not available in our healthy control group. The reason for that is that we got no permission from our ethics committee to perform lumbar puncture in healthy controls without any signs of cognitive impairment. As the prevalence of preclinical AD among cognitively healthy elderly people is estimated to be about 20%, we cannot exclude the same prevalence for our cohort (Parnetti et al., 2019). Therefore, our findings should be replicated in an independent cohort including healthy controls without signs of AD pathology. Third, even though we found no statistically significant influence of antidepressant use (*p* = 0.25) and AChE inhibitor use (*p* = 0.21) on the relative abundance of genera, taxonomic and functional differences between the AD patients and healthy controls may have been influenced by drug exposure. Furthermore, we measured no metabolites in the collected gut samples.

In conclusion, we showed that intestinal microbiome can be used to discriminate amyloid-positive AD patients from healthy controls. The diagnostic accuracy increases from taxonomic to functional data and is even better when combining taxonomic, functional and clinical models. Intestinal microbiome represents a promising area for developing novel interventions against AD.

## Data Availability Statement

The datasets presented in this study can be found in online repositories. The names of the repository/repositories and accession number(s) can be found below: https://www.ebi.ac.uk/ena, PRJEB47976.

## Ethics Statement

The studies involving human participants were reviewed and approved by Institut für Ethik und Geschichte der Medizin, Gartenstr. 47, 72074 Tübingen. The patients/participants provided their written informed consent to participate in this study.

## Author Contributions

CL and MW participated in study concept and design. CL, SM, OP, VR, MM, SP, US, and MW participated in the acquisition, analysis, and interpretation of data, and in the critical revision of the manuscript. CL drafted the manuscript. US did the statistical analysis. All authors read and approved the submitted version of the manuscript.

## Conflict of Interest

The authors declare that the research was conducted in the absence of any commercial or financial relationships that could be construed as a potential conflict of interest.

## Publisher’s Note

All claims expressed in this article are solely those of the authors and do not necessarily represent those of their affiliated organizations, or those of the publisher, the editors and the reviewers. Any product that may be evaluated in this article, or claim that may be made by its manufacturer, is not guaranteed or endorsed by the publisher.

## Footnotes

1
https://www.bioinformatics.babraham.ac.uk/projects/fastqc/

2
https://github.com/biobakery/kneaddata

3
https://www.genome.jp/kegg/pathway.html

4
http://geneontology.org/
