# Supplementary material for: Signature of Alzheimer’s Disease in Intestinal Microbiome: Results From the AlzBiom Study
Source: Front Neurosci. 2022 Apr 19;16:792996. doi: 10.3389/fnins.2022.792996 (PMC9063165; doi:10.3389/fnins.2022.792996)
Supplement: Supplementary file 1 [file Data_Sheet_1.pdf]

Supplement to "Alzheimer signature in intestinal microbiome: Results from the AlzBiom study."

Ulrich Schoppmeier

November 24, 2021

Abstract

The paper "Alzheimer signature in intestinal microbiome" presents several findings about the connection of bacterial microbiome composition and Alzheimer's disease. The key problem was to find an appropriate classifier based on the microbiome data and additional clinical and biometrical information.

In this text details about the preprocessing of data and data analysis are presented. The statistical modelling was performed by application of logistic regression to normalized data under the paradigm of compositional data analysis. This relies heavily on the use of balances and preselection of features. As several different aspects of microbiome features and clinical as well as biometrical data resulted in a variety of models, these models were combined later by ensemble learning.

Contents

1 Introduction. 3

2 Description of Data. 4

2.1 Data Types 4
2.2 Data Format 5
2.3 Data Sources 5

3 Preprocessing. 5

3.1 Introduction. 5
3.2 Selection of Cases due to the Outcomes. 6
3.3 Separation in Training and Validation Data Sets. 6
3.4 Data Cleaning and Imputation. 7
3.5 Normalisation. 8

4 Data Analysis. **9**
4.1 Introduction. 9
4.2 Wilcoxon's Tests' p-Values. 9
4.3 Number of Outliers. 10
4.4 Quantiles. 13
4.5 Other Statistical Analysis. 13

5 Statistical Model Building. **14**
5.1 Introduction. 14
5.2 Balances. 16
5.3 Logistic Regression. 19
5.3.1 Balances and Logistic Regression. 20
5.3.2 Preselection of Features. 22
5.3.3 Feature Selection Process. 23
5.3.4 Validation. 24
5.3.5 Results. 25
5.4 SELBAL. 27
5.5 Ensemble Learning. 27
5.5.1 Results. 28

1 Introduction.

The task was to create a model for prediction of Alzheimer status from information about bacterial microbiome compositions. The Alzheimer status was coded as a binary outcome (disease versus no disease). Several types of information about the microbiome data referring to different aspects were available in abundance tables (details are given later). In addition, we used clinical data (age at sampling, APOE, body mass index and gender).
We intended to create predictive models for the outcome based on microbiome and clinical metadata. After some attempts employing different types of machine learning approaches we decided to use logistic regression. As the data had many features, we applied feature selection. Three different types of selection were employed. Firstly, we observed, that feature with too many outliers were potentially harmful for a stable model. So we selected features accordingly. Second, we ran a Wilcoxon test and used the p-values in a preselection of features. Third, we designed a feature selection using the logistic regressions: Starting the modelling with a certain group of preselected features, the features were reduced step by step by discarding the worst one. The assessment of models was achieved by the calculation of the mean accuracy in a cross-validation approach. This resulted in a series of models each having one feature less. In each of these series we identified the best model (again using the mean accuracy as measure).
At an early stage of our analysis we divided the data into training and validation data sets. For all data analysis and model training issues only the training data were used. A crucial step in the model training was to find some good and general preselection rules. Assessing the rules by validation data must be avoided! Checking box plots of features was helpful. Besides model quality another benefit of reducing the features was the acceleration of run time.
As the abundance tables were compositional data, compositional data analysis was used as described later. We want to emphasize that using the concept of balances was crucial. Balances allow to condense many features of a composition to just one number. This helps in connection with logistic regression.
The variety of data types resulted in several different models. One group of models used only the different types of microbiome data, another one the microbiome data together with the clinical data and the third one only the clinical data.
These models were assessed on the validation data. Finally, we merged several of these models by ensemble learning to improve the model quality.
All statistical calculations were made by employing a lot of *R* code. This code was written by the author using many existing libraries. The code will be made available on a specific repository. There we will give the references to the libraries' authors.

2 Description of Data.

2.1 Data Types

The data could be categorized roughly as clinical metadata and microbiome data.
Metadata contained 175 data sets about participants. There were 20 features given for each data set. Only a part of this is included as outcome or as clinical data respectively into the analysis. The data fields used are shown in the next table.

ID
DNAID
Alter
Geschlecht w1/m2
APOE4
BMI
Amyloid-positiv (1) vs. Gesund (0)

Table 1: Data fields of relevance from metadata file (German names of data fields are displayed).

In some cases information was missing for APOE and BMI. We applied methods of imputation to overcome this.
The microbiome data can be classified following the scheme:

- taxonomic information (or *otu*):
  - phyla
  - genera
  - species
- functional information from matching with:
  - KO data base
  - GO data base
  - EGGNOG data base

The distinct features in taxonomic information are also known as *operational taxonomic units* (short *otu*).
These information on microbiome composition were provided in separate tables. Any type of these data constitute a distinguished aspect of microbiome composition. Mixing these information might result in redundancies. Only at the step of ensemble learning models were built using information of different aspects.
Due to the nature of microbiomes in many cases entries were either zero or lacking. This raises problems in the course of compositional data analysis. Methods for imputation are discussed in the sequel.

2.2 Data Format

The metadata were supplied in an Excel file spread over several sheets. Problems were encountered with some of the column headers. But it was easy to cope with that.
Microbiome data were supplied in Excel or in csv format. Difficulties with these data arose due to different way to express missing data: in some cases a 0 was used in others the fields were left blank.

2.3 Data Sources

Metadata were collected under the supervision of the study nurse in charge Iris Honold and of Prof. Dr. Christian Laske (both at the Department of Psychiatry and Psychotherapy in Tuebingen). The information was gathered in an Excel file. For this investigation only a small part of the data is of concern.
The microbiome count data were provided by Prof. Dr. Matthias Willmann (Formerly Institute of Medical Microbiology in Tuebingen, now at Eurofins Medical Lab Gelsenkirchen in Gelsenkirchen) in several files:

- `AD_Taxo_Spezies_ALL.txt`:
  300 data sets of 3 078 fields.
- `Alzheimer_Genus_All.xlsx`:
  300 data sets of 1 019 fields.
- `Alzheimer_Phylum_All.xlsx`:
  300 data sets of 43 fields.
- `All_EGGNOG_Abundances.xlsx`:
  300 data sets of 15 213 fields.
- `All_KO_Abundances.xlsx`:
  300 data sets of 6 444 fields.
- `All_GO_Abundances.xlsx`:
  300 data sets of 9 791 fields.

Again as in the case of the metadata only a part of the data sets is covered in this analysis.

3 Preprocessing.

3.1 Introduction.

Preprocessing in a vital step in modelling. As the titles of the following subsections demonstrate, there were several different tasks: Some were about merging the information supplied by metadata and by microbiome data. A problem here

was that different keys were used in the identification of samples and participants. Another decisive step was the separation of training and validation data. In fact as we first planed to use microbiome data and outcome only (not considering other metadata), we did this step very early in the project. We did not change the assignment when we decided to take further metadata into account. Analysing the data in the spirit of compositional data analysis involves logarithms of feature abundance data. Missing data or even zeros cause errors here. So we had to find a reasonable method for imputation. Imputation was also needed in case of some metadata. During the modelling we looked for the consequences as some of these data were used as predictors in the models. Luckily these predictors were not very influential.
The calculations and data manipulations were done using *R* and *HeidiSQL*. It was very fortunate to hold the data in a data base, and *HeidiSQL* worked fine with *R*. So generally we loaded data from *HeidiSQL* into *R*, manipulated them there and wrote the results back to tables in the data base.

3.2 Selection of Cases due to the Outcomes.

We are interested to model the status of Alzheimer's disease. In the original survey there were three distinct groups: healthy, patients with Alzheimer's disease and those with mild cognitive impariment. Each groups contained 100 participants. Only the first two groups are of concern here. In order to have a precise definition of the status "ill" we decided to include only those patients biologically from the second group which had a positive result of amyloid protein detected in their cerebrospinal fluid.

outcome	0	1
# data sets	100	76

Table 2: Values of field `Amyloid-positiv (1) vs. Gesund (0)` before removing one data set.

One data set had to be removed. The status of this participant was "Amyloid-positiv". This is coded in the accompanying *R* script.

outcome	0	1
# data sets	100	75

Table 3: Values of field `Amyloid-positiv (1) vs. Gesund (0)` after removing one data set.

3.3 Separation in Training and Validation Data Sets.

There's one distinct script for the creation of training data and validation data indices. This script written by the author relies on the `caret` library of *R* [9]. The split is 3:1 respecting as much as possible the frequencies of outcomes. In

this way we can guarantee the same cases to be selected for the different otu and functional aspects. Irrespective of the phyla, genera, species, KO, GO or EGGNOG aspect the same cases were allocated to training and validation data sets.
Processing the assignment of the cases to the training and validation data subsets it is important that the (relative) frequencies of the outcome groups should be similar. Due to the integrity of counts this is not perfectly possible.

Training			Validation			
0	1	0:1	0	1	0:1	Tr/Val
73	59	1.24	27	16	1.69	3.07

Table 4: Outcome's frequencies in training data and validation data.

3.4 Data Cleaning and Imputation.

The issue of missing data concerns the microbiome data and the clinical or biometrical data.
Absence of data in case of APEO and that of body mass index (BMI) is often due to problems with the compliance of participants. For the APOE status blood from the participants is needed. We used hot deck imputation for coping with missing data:

- The data sets with known APOE status were selected.
- For each data set lacking the APOE status one instance with known status was drawn by chance (sampling with replacement).
- Imputation was carried out using the drawn sample.

This hot deck imputation does not change the expected frequencies of APOE status.
In three cases of BMI we got no information either about weight or height of the participants. Even in this case we used hot deck imputation. It seemed advisable distinguish the instances by gender.
Missing data in the microbial ralative abundance tables show up in two manners. Either the entry is empty or it is zero. At at each stage of the sampling there is a certain possibility that components of the microbiome might slip through the detection. It even might be possible for some species to be present only in some samples.
Lacking entries or zeros in the count data make trouble in the analysis as we will after appropriate normalisation look at logarithmic count data. Empty entries are troublesome for normalisation and zeros prevent the application of logarithmic transformation.
Our working hypothesis here is that missing data are due to low relative abundance so that it falls below the detection limit. In connection with compositional data one might consult for example [16] about classification of reasons for missing data.

As imputation is for us a technical matter, we decided to determine the lowest count c_{aspect} in the training data. Then we fix the detection limit as $c_{aspect}/1000$. All zeros and missing values in training and in validation data were substituted by this value.

Aspect	lowest value c_{aspect}
Phyla	0.022
Genera	0.017
Species	0.017
GO	0.004
KO	0.003
EGGNOG	0.004

Table 5: Lowest relative abundance values in the training data different from 0.

If ever new data would be used one must take these figures as constants for the imputation.

3.5 Normalisation.

Later we emploed the concept of *balances* which is connected with compositional data analysis. An introduction to the ideas of compositional data analysis is given in [1] and [2]. [16] and [5] review some applications in connection with *R*. A short introduction and some general remarks on applications can be found in [7] whereas [10] presents an introduction to the statistical theory. In connection with microbiome data [6] and [14] should be mentioned.

From all the ideas of compositional data analysis we only use balances here in connection with logistic regression. We need our microbiome data to be normalised in the manner that the relative abundances of all features in a data set sum to a constant – commonly taken to be 1.

Performing the normalisation is quite easy. For each data set $x_0, x_1, ..., x_n$ the positions of features have to be identified as the meta information on outcome must not be involved in the normalisation. Let's say x_0 contains the outcome. Then the normalisation factor is simply $1/\sum_{i=1}^{n} x_i$ so that the new features are $x_k/\sum_{i=1}^{n} x_i$ for $1 \leq k \leq n$.

Obviously any metadata have to be excluded in that calculation. Additionally there are sometimes features in the microbiome data, that we do not want to consider (eg viruses or unclassified features). These were caused by reads, that could not be assigned properly to any otus or functional information in the data bases and excluded from normalization and further analysis also.

4 Data Analysis.

4.1 Introduction.

In this introduction we will explain our efforts in data analysis. Merely they serve as a preparation for the logistic regression. First we would like to define some terms. We useed *response* and *outcome* interchangeably for the data to be modelled. The data involved in the model formulas are called *explanatory variables*, *regressors*, *predictors* or *covariates*. But we try to restrict the later for categorical data. These terms are used in connection with the models. In connection with data fields in tables we use *outcome* and *feature*. So we have two distinct types of features, those which steam from microbiome relative abundance tables and others from metadata. *Features* do not enter directly in our logistic models unless they are *covariates*. For instance relative abundance data only encounter the logistic regression functions via the *balance functions*.
The *balance functions* or balances play a decisive role in our approach. Grace to the balances a condensation of information about the microbiome data is possible. In consequence the logistic regression need not to cope with many explanatory variables. On the other hand a good selection of features entering the balances is vital for a good model showing strong capability of prediction.
The model training comprises some kind of feature selection to be discussed later. For computational reasons and to insure good performance even on the validation data, a preselection of suitable features is indicated. We decided to combine two different approaches. The first one is based on the p-values of a two sample Wilcoxon test. It should be emphasised that we only use the p-values for sieving the features. Details will be given later.
The second approach came into our minds when looking at box plots of some data. We were in doubt whether features with too many outliers or with very large "boxes" absorbing any outliers might be useful. So we sought for some criterion confining the spread of data. Variance or interquartile distance comes immediately to ones mind. But we couldn't manage to find a general rule about these indicators. At the end we decided to exclude features having too many outliers and those having no outlier. Perhaps the last criterion might seem odd. In fact it excludes features that were missing in too many cases. These features might be called *unsuitable*.
One should add that we did these analysis only for the training data as we regard this step as a part of the training.
For sake of completeness we present some additional statistical analysis that serve for general purposes in the course of the survey.

4.2 Wilcoxon's Tests' p-Values.

Table 6 shows the a small part of the normalized abundance data on species for the medians of outcome groups 0 and 1 in ‰ (in *pro million* for the first line). The first five lines are the data according to the five highest p-values. Last lines are that of the five smallest p-values. It is quite understandable that one is

only interested in features, that show remarkable differences between the two outcome groups. A common way to select interesting features in these situations is to apply some hypothesis test for difference between the groups, calculate the p-values, order the data accordingly and just sieve out a part of the data. As we the distributions are probably fare from normal distributions we applied the Wilcoxon test.

Taxum	median of group in ‰	
	0	1
Actinoplanes sp SE50	0.00004	0.00005
Calothrix sp NIES 4071	0.0236	0.0248
CandidatusNitrosoglobus terrae	0.0124	0.0127
Chania multitudinisentens	0.0099	0.0099
Dactylococcopsis salina	0.0142	0.0156
Acidovorax sp NA2	0.0003	0.0006
Erwinia pyrifoliae	0.0016	0.0009
Bartonella sp JB63	0.0004	0.0002
Escherichia fergusonii	0.0075	0.0061
Pseudomonas mandelii	0.0018	0.0036

Table 6: Medians of microbiome relative abundance table on species. Shown are the features having the five highest and the five lowest p-values. Medians are of ‰exept for the first line were it is in per million.

As we employed the standard implementation of Wicoxon's test in R there's not much to say: For each normalised feature at hand we separated the training data values according to the outcome into two groups. A two-sided Wilcoxon test for two samples was performed and the p-value extracted from the output. Later in the model training we selected an appropriate number of features by varying the threshold. The threshold was adjusted so that we got enough features and the choices varied. Details are given later.

4.3 Number of Outliers.

Outliers were defined in accordance to Tukey's proposition in [15]: Let $(f_{i.k})_{1\leq k\leq K}$ the normalized abundances for the ith feature (the index k runs from 1 to K through the case indices). We will later on work with logarithms of normalized abundances. This is due to avoid computational problems in connection with balances. Therefore the outlier detection and quantile analysis rests on logarithms. The quartiles q_1, q_2 and q_3 of the logarithmic normalized abundances are the 25%, 50% and 75% quantiles of the data (we suppress here any reference to the index i or the specific feature). q_2 is also known as the *median*. The difference $q_3 - q_1$ is called the *interquartile range* (short *IQR*). This information is visualised in a *box plot*. For example look at figure 1 where we show an example. The vertical position of the central box is defined by the two quartiles q_1 and q_3. The boxes vertical extension amounts to the interquartile range so

that it represents 50% of the data. In the box a bold line marks the median's position. The vertical lines resemble the other data as long as they are

- either fall between $q_1 - 1.5 \times IQR$ and q_1 for the lower whisker
- or fall between q_3 and $q_3 + 1.5 \times IQR$ for the upper whisker.

This means that a whisker can be of length $1.5 \times IQR$ at most. In the figure 1 we marked the values $q_1 - 1.5 \times IQR$ and $q_3 + 1.5 \times IQR$ by dashed lines. No data are larger than $q_3 + 1.5 \times IQR$. At the other end there is one data point which is smaller than $q_1 - 1.5 \times IQR$. This is by rule assigned to be an *outlier*. It is marked as a bold point in the box plot.

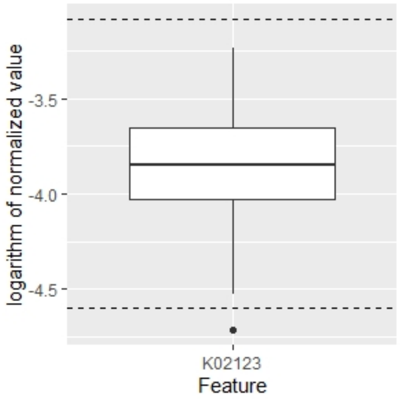

Figure 1: Example of a box plot.

We recalled here the construction of box plots and that of the outliers' definition because in our analysis we employed both concepts many times.
In the code the outliers were identified automatically and counted. These numbers along with information about quartiles were reported in a text file.
Now we give a short discussion of the role of outliers in the prediction problem. Figure 2 depicts some data on features. These data were split into the two outcome groups. The Wicoxon test's p-values were below 0.006. At first these features might seem to be good candidates for establishing a predictor. In fact they have

- either to many outliers as in the second row
- or the interquartile range is large and no outlier can be identified (as in case of the feature K01849).

Both situations are bad for good predictors as outliers as well as large interquartile ranges indicate shaky information.

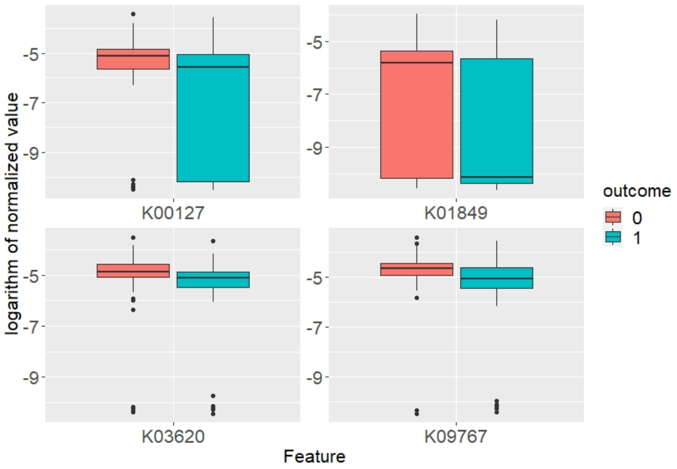

Figure 2: Features with either many outliers or large interquartile ranges. Only training data were considered.

The problem of large interquartile ranges was difficult to address. We decided to apply a rule about maximal and minimal numbers of outliers in the data. The specific rules will be given later as they depend on the type of data.

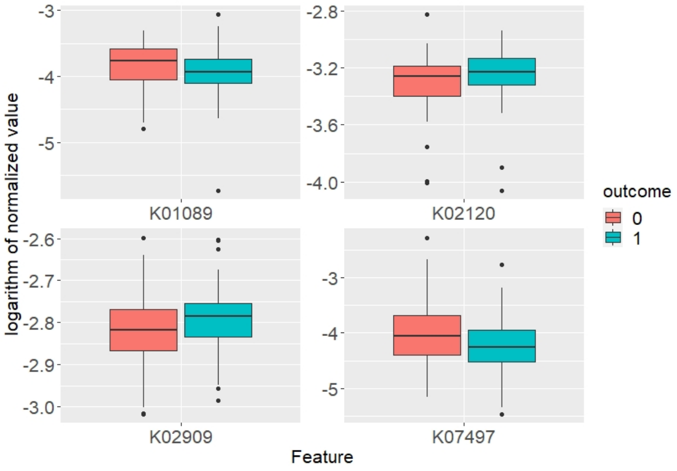

Figure 3: Features with nice behaviour regrading outliers and interquartile ranges. Only training data were considered.

Figure 3 shows some nice features. Mark that the medians are different between the two outcome groups. This is an indication that they will be suitable for model building purpose.

Finally we want to mention that large interquartile ranges might be caused by to many instances of imputation.

4.4 Quantiles.

In addition we calculated several other values for descriptive statistical use like medians, other quartiles or interquartile ranges. Some of the quartiles were occasionally used for feature preselection also.

4.5 Other Statistical Analysis.

To show the relationship between some clinical data and the features we evaluated the Pearson correlation. The features were limited to the set selected during the model training step. The results are shown in figure 4 as heat maps. In addition to that we determined the p-values in a correlation test and corrected for multiple testing with Bonferroni's method.

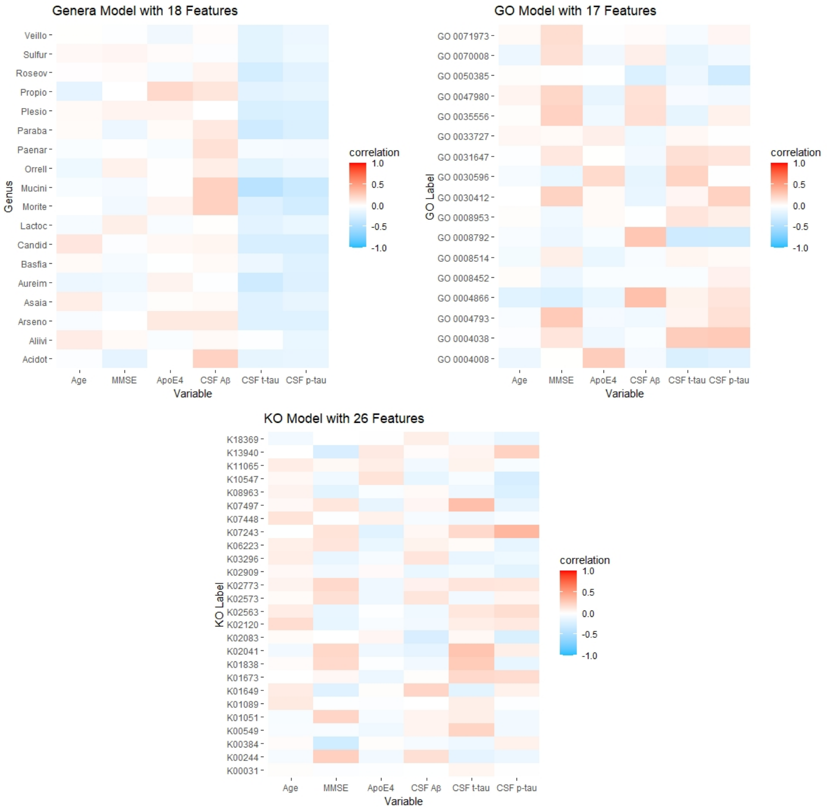

Figure 4: Heat maps of Pearson correlation coefficients for some clinical data with selected features.

Interestingly for one variable (CSF p-tau) in case of KO data there seemed to be a significance. In fact that was due to a typo in this clinical data field.

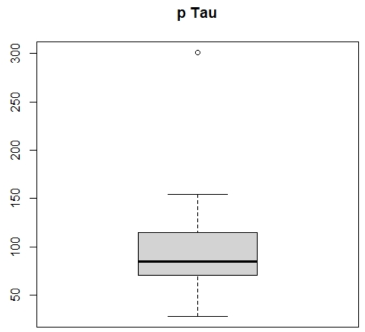

Figure 5: Typo in the data field CSF p-tau identified as outlier in a box plot.

As for many participants these clinical data were not available, the information shown in the heat maps is for information only.

5 Statistical Model Building.

5.1 Introduction.

We seek for a model which might loosely be written as

$$outcome \sim microbiome\ features + clinical\ data$$

Here the data field *outcome* is binary, microbiome features are numerical or more precisely compositional and clinical or biometrical data either nominal (gender), ordinal (APOE) or numerical (BMI). The number of features in microbiome data ranges from about 1 000 to more than 15 000. This would render regression methods impossible if features enter as explanatory variables into the model directly. Luckily three different types of remedies are at hand. First of all we do not use the microbiome features as explanatory variables. Instead we use a certain function of the features called the *balance*. This data reduction shrinks the number of explanatory variables considerably. But there is a certain drawback: Whereas in regression models the regression coefficients might give some information about importance of explanatory variables, this lacks when balances are used. So one has to find another way to separate statistical information from noise. And this is reached by two other remedies: Preselection of features and a feature selection during the model learning phase.
Preselection has at least two merits. The first one is plainly to speed-up the calculations by reducing the amount of data. The second one is connected

with stabilisation of the model. We seek for features which add to the model's classification power. We must prevent features entering the model, that are different for the two outcome classes only by chance. They would probably give nice behaviour of the model for the training data but bad on the validation data and perhaps worse on new data. If they behave badly on the validation data, one would have the chance to detect the problem. But this is not guaranteed and to late for sound model building. Mind that validation data are reserved for final the assessment of a model. If one removes bad features identified in the assessment of a model using the validation data this is a part of model learning! The validation data would be worthless for further assessment. Better to find a general rule for excluding these unsuitable features before seeing the validation data.
The last remedy used here is feature selection during the training phase. This is a common approach. Unfortunately starting with a unsuitable selection of features might give poor results even with sophisticated feature selection. One should have in mind that we have a very limited number of cases parted into training and to validation data. The power of the resulting small set of training data to "identify" nice features might be poor. So we should "help" by preselection and application of general rules.
Another thing to mention is that we have six different types of microbiome data and four clinical data. There are three types of models possible:

$$outcome \sim balance\ of\ microbiome\ features$$
$$outcome \sim balance\ of\ microbiome\ features + clinical\ data$$
$$outcome \sim clinical\ data$$

This makes $6 * 2 + 1 = 13$ different possibilities to combine the data types for modelling (we never intended to mix different types of microbiome data in one model at this stage). Although it is at least possible to investigate the models in an ANOVA manner, we dropped that. Instead we compared our final models in terms of AUROC values (see below). These assessments were performed on the training and on the validation data. Results are presented later. At the end we combined some of the models by *ensemble learning*.
In the next section we talk about balances, then on generalized linear models and how we employed these to solve our problem. We should mention here that our analysis owes much to the *SELBAL* project (cf. [14]). Although we did not use the SELBAL algorithm for modelling, we got at least the idea of balances from this paper. In fact our first attempt was to implement a feature selection using the features as explanatory variables directly instead of the balance. The results were disappointing. Then we tried SELBAL with promising outcome. Assessing the resulting models on the validation data was a bit frustrating. So we put together our feature selection process and the balance concept to gain a promising trail.

5.2 Balances.

Balances were introduced by Vera Pawlowsky-Glahn and Juan José Egozcue in [4]. To explain the idea of balances in models on compositional data, we begin with a very simple example. Let us look at figure 6. There is one feature and we want to classify the two outcome groups using this information only. Regardless of any details about the specific modelling approach to be used, we should be happy if the red box representing the mid 50% of the data in the outcome group 0 does not to much overlap with the blue one representing the mid 50% of data in group 1. At least we would like the medians of both groups to be well separated.

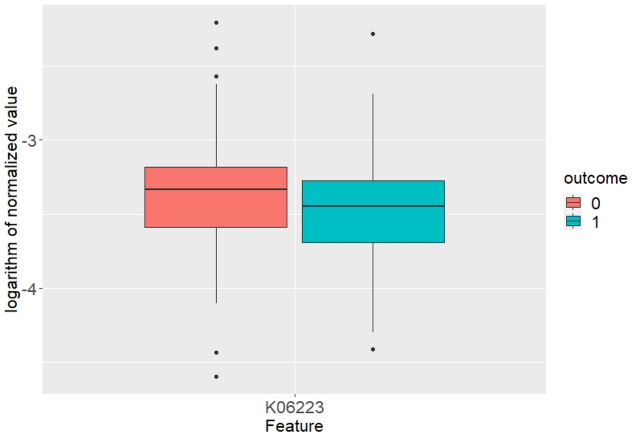

Figure 6: Simpe example for the explanation of a balance.

This picture resembles scales or a *balance* and we want the two scale pans (either the boxes or the medians) to be as far from equilibrium as possible. Now having more than just one feature (as in figure 7) one should try to find suitable features and combine them. As shown in figure 7 there are quite effective features. But they might move the balance to different directions. In effect they might compensate their action. The best remedy is to adjust the sign of the data.

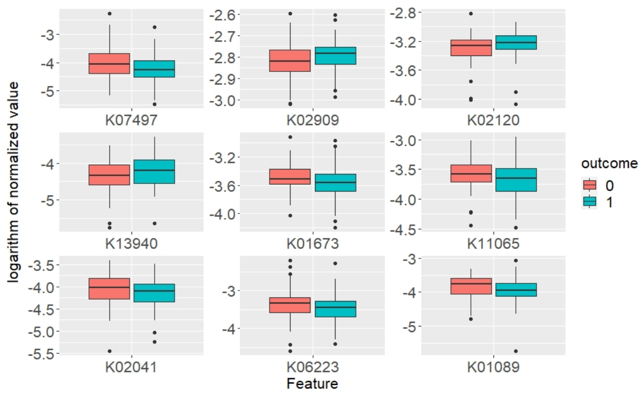

Figure 7: Examples of Features. Normalized data were plotted after taking logarithms. Numbers in labels are the p-values of a Wilcoxon test. Only training data were considered.

Gradually we approach the idea of balance functions. When thinking of putting weights to the scale pans, in fact we *add* something. For our problem it would be tempting to just add all the features on one side after adjusting the signs properly. This would be a too easy solution!
Instead we have to adjust for the number of features involved: We have to take averages! For compositional data arithmetic averages are a poor choice. Better is to take geometric averages. This interferes badly with our idea of adjusting signs. So let's take logarithms. Then arithmetic averages are perfectly justified. After this preparation we present some formulas.
Again let $f_{i,k}$ is the normalized abundance of feature i in k^{th} participant's microbiome. To have an example at hand look at figure 7. For the balance we would assign the features into two categories depending on the relative position of medians in the two outcome groups. The specific names of these categories do not matter! Just to comply with the literature we use the terms *Numerator Category* and *Denominator Category*.
In our example we get the following assignment:

- Numerator Category:
  K07497, K01673, K11065, K02041, K06223, K01089

- Denominator Category:
  K02909, K02120, K13940

We might even define the categories in the opposite manner. All that matters is that the difference in the medians between the two outcome groups in the numerator category have the same sign and the opposite sign in the denominator category.
Let be N the numerator category and D the denominator category. $\#N$ and $\#D$ are the numbers of features in theses category being 6 and 3 in our example.

Then the geometric means after adjusting the signs and some simple algebra result in the expression

$$B(k) = \frac{\sum\limits_{i\in N} \log(f_{i,k})}{\#N} - \frac{\sum\limits_{i\in D} \log(f_{i,k})}{\#D}$$

for the value of the balance function given the features and k^{th} participant. To sum up the value of a balance is the arithmetic mean of logarithms of normalized features abundances after suitable adjustment of the signs.
After doing this calculation for all participants we can plot the resulting balance values again in a box-whisker plot. This is done on the left hand of figure 8. Mind that we just took some features without any fancy feature selection in the example. A nice result from real-life is shown on the right of that figure. Now the boxes are nicely separated. Features selections like this are promising for further use in logistic regression analysis. We will explain that below.

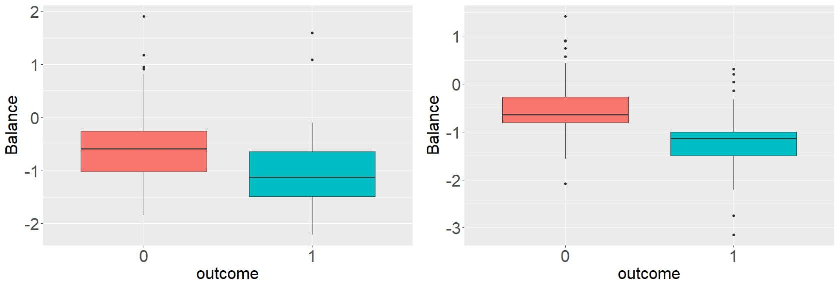

Figure 8: Balances calculated for the two outcome groups. Left the nine features shown in figure 7 were used. The effect of combining features to a balance is remarkable: The two boxes indicate 50% of the data. Their overlap is smaller than for example that shown in figure 6. On the right the balances used in the best model found are given. 26 features were used. The distributions of balance values for the two outcome groups differ: The boxes do not overlap! The logistic regression function calculated with these data is plotted in figure 9.

We should comment here on the consequence of taking logarithms. Defining the balances in this way using logarithms and their arithmetic averages avoids computational problems. Computers do not like division by small numbers. Without taking logarithms the definition of a balance would be

$$b(k) = \frac{\sqrt[N]{\prod\limits_{i\in N} f_{i,k}}}{\sqrt[D]{\prod\limits_{i\in D} f_{i,k}}}$$

and the relation $B(k) = \log b(k)$ holds. As soon as the denominator gets close to zero trouble lurks around the corner. In the expression for the denominator

even some terms close to zero are dangerous. This is avoided with arithmetic means. But there we have to take logarithms. This is not that dangerous if one keeps the value 0 away (there's no logarithm of 0). That is the reason why we bothered with imputation in case of microbiome data.

5.3 Logistic Regression.

Logistic Regression will serve here for building a classifier. References are [8], [12] and [3] for example. In the case of using the balances the model can be written as

$$P(k's\ Outcome = 1 \mid b_k) = \frac{\exp(b_k\beta)}{1+\exp(b_k\beta)}$$

Here b_k symbolizes the balance's value for k^{th} participant's microbiome and β stands for the regression coefficients. The function defined by

$$f(x) := \frac{\exp(x)}{1+\exp(x)}$$

is known as the *logistic function.* The black solid curve in figure 9 shows a typical shape. The task of *logistic regression fitting* is to estimate the form of this function best.

In some models we will include additional explanatory variables. Depending on whether these are of categorical or continuous type, the model expression has to be altered in different manners. Luckily using *R* there's no trouble. We employed the `mlr` and the `stats` library for model fitting.

One should have im mind, that in contrast to linear regression there's no closed formula for a solution in case of logistic regression. The calculation of the coefficients rests on numerical search algorithms. Start values are needed for initialization here. The methods implemented in *R* do all the work in the back round. But the user should be aware that results might change a bit from run to run.

Just to make our use of terminology clear again:

- The terms *outcome* and *response* or *response variable* are used as synonyms here. We prefer *outcome. Dependent variable* will never be used.
- *Regressor*, *predictor*, *predictor variable* or *explanatory variable* is the name for the generic data entering the model by the regression formula. So our predictors are balances, gender, age, BMI, APOE respectively.
- The word *feature* is reserved for the data characterizing the microbiome composition in other words the abundances of phyla, genera etc.

In the next section we will explain in detail how the logistic regression together with the balance serves as a classifier.

5.3.1 Balances and Logistic Regression.

In figure 9 we present an example of the interplay between balances and logistic regression. The balance is based on a suitable selected set of features. The balance's values for the individual data sets are plotted as blue or red dots parallel to the x-axis. The positive cases having outcome 1 are in blue and the negatives in red. The dots are placed according to the outcomes and the balance's values.
Fitting a logistic curve via logistic regression to these data, we come to the curve plotted in black. The evaluation of the logistic curves for the balance values results in the blue and red dots printed parallel to the y-axis. These are the predicted probabilities to be in the outcome group 1 given the balance's value. Instead of using the term *predicted probabilites* we prefer *predicted likeliness* here.
Yet the predicted likeliness is not the classification. The last step is to decide upon a threshold θ so that

- if the predicted likeliness of a case is larger than θ, the case is assigned to the outcome group 1,
- else it is classified as being a member of outcome group 0.

In the example the threshold $\theta = 0.50$ is depicted as a dashed horizontal line. Now we can count the number of misclassification based on this threshold. There are 6 false positives and 10 false negatives. Whether this is acceptable depends on the user's attitude. If one wants to avoid any false positives, one might chose $\theta = 0.95$. At least in case of our training data there would be no false positives – but many false negatives. This example makes clear that there is a trade off between reducing the number of one misclassification at the expense of the other.

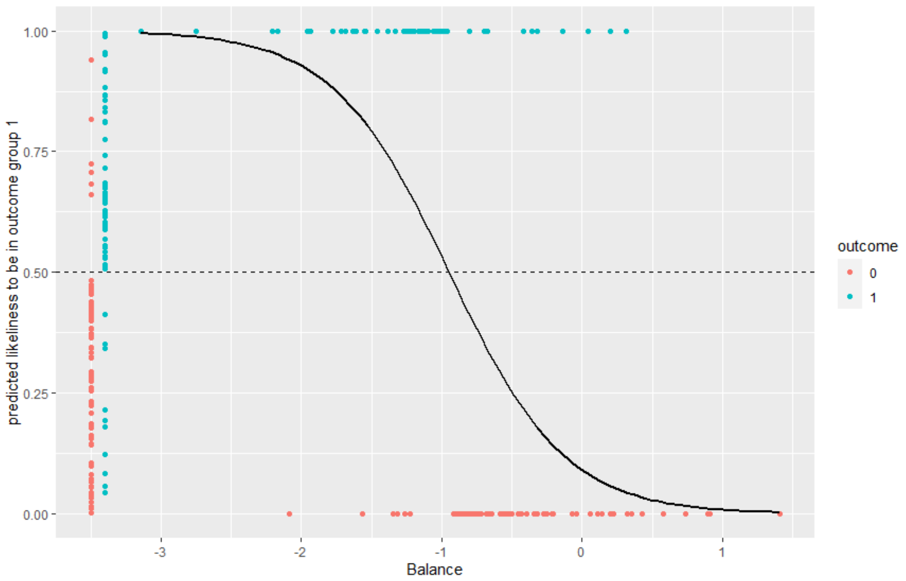

Figure 9: Example of a logistic regression. The logistic function (black solid curve) was fitted to the balance, which is shown in figure 8 on the right. Blue or red dots parallel to x-axis indicate the values of the participants' balances grouped by outcome. Blue or red dots parallel to the y-axis mark the predicted likeliness to be of outcome 1 (or positive) for each participant. The model was fitted to training data and only these data are shown. The threshold for decision rule might vary on y-axis giving different numbers misclassification errors. For the threshold of 0.5 indicated as dotted black line one gets 10 false negatives and 6 false positives.

By the means of *ROC curves* one can get an impression about the influence of the threshold on the misclassification. In this type of visualisation the *false positive fraction* and the *true positive fraction* as measures of misclassification are plotted against each other. These two numbers were evaluated for each choice of $0 \leq \theta \leq 1$. The threshold θ is not plotted in this diagram! The jumps are due to the finite number of cases in the training data set. This results in an only discrete number of different *false positive fraction* and *true positive fraction* combinations. The resulting points were connected by straight lines in the diagram. In fact these ROC curves are only estimators for the model's ROC curve!
These (estimated) ROC curves can serve for a model's assessment. The best model would simply result in a false positive fraction 0 and a true positive fraction of 1 for any choice of $0 < \theta < 1$. This gives the straight horizontal line $true\ positive\ fraction = constant = 1$. The overall quality of a classification model is then measured by the *area under the ROC curve* (AUROC). For the best model one gets an AUROC of 1.

It should be emphasized, that assessing the (trained) models on the data used for training (the *training data*) is not sufficient. A well trained model would give good results on the training data although it might not generalize the information contained in the data. It is best practice to assess the models on hold-out-data or *validation data* that were never used in any step of model training!

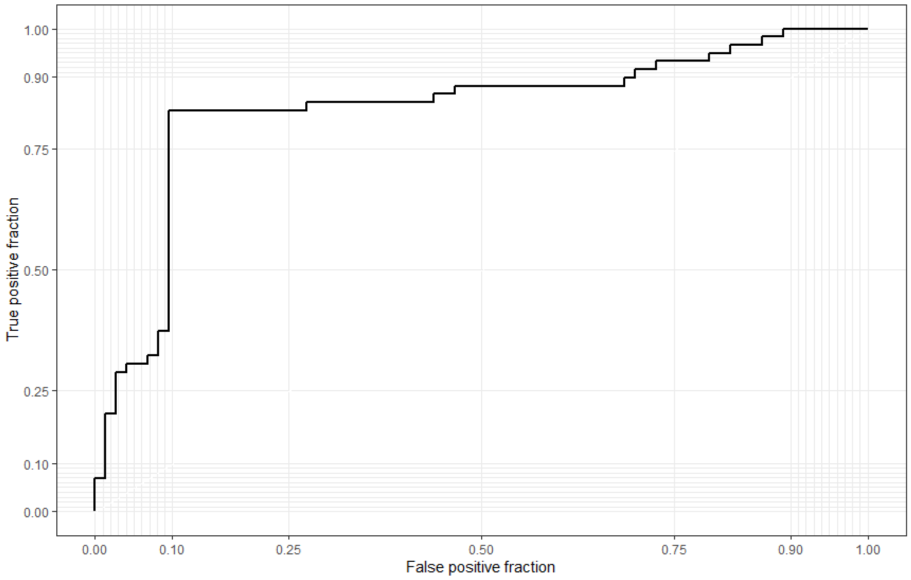

Figure 10: Example of a ROC curve. The ROC curve was calculated for the model gained by the logistic regression example in figure 9. Only the training data for assessment were used for the assessment here. Notably one should assess the model also on the validation data.

We preferred AUROC for assessing our models. The values are presented in the results. Certainly many different *R* functions and libraries were employed and we owe much to the their developers. Detailed references will be given in the code documentation.

5.3.2 Preselection of Features.

We mentioned before the reasons for preselection of the features:

- avoiding bad models that incorporate features with too many outliers of large interquartile ranges.
- speeding up computing time
- reduction of data volume

As there were up to more than 15 000 features in the data, a step-wise feature selection would be unfeasible.
Several rules were applied in combination to gain a suitable subset. The p-values of the Wilcoxon test mentioned before were subjected to a sieving. Certain thresholds were applied ranging between 0.07 and 0.5. We mention this range here to express again that the Wilcoxon test must not be interpreted in the spirit of classical hypothesis testing here. Therefore we could relinquish on any provisions for multiple testing.
Another rule was to demand for at least one outlier and at most 2 or 5 or 6. The specific choice depended on the data.
The last selection criterion was about the 25% quartiles. With this we wanted to prevent features with too many instances of imputed values in the preselected feature set. In fact either the rule about the minimal number of outliers or that about the 25% quartiles was combined with the criteria about the maximal number of outliers and the p-value. Details will be given in the results.
The draw back of preselection is that possibly some interesting features might not have the chance to ever enter the model. We tried to avoid that by using quite liberal rules. We even looked at some excluded features whether we missed something. But we never found any obvious flaws.
The resulting selection was assessed by

- Box plots of balance values based on the training data. There should be a considerable distance between the medians and the two boxes in the plots concerning the two case groups. Additionally the two boxes should not overlap completely.
- Box plots of some features. This was done only when finding good rules was difficult. The drawback with this approach is the large number of plots when this analysis was applied to all features in a preselected set.
- Finally the preselected feature set was used for training and the AUROC was assessed. If this was too low (below 0.7 let's say), the selection was discarded.

As we were not that happy with that the try and error approach we attempted to develop a new approach by bootstrapping the medians on the training data split by outcome. We postpone that idea.

5.3.3 Feature Selection Process.

Starting with a set of N features, N different balances were calculated each containing $N - 1$ features. Any of the features in the starting set was left out exactly in one balance.
For each balance the training sets were then parted in five portions randomly. In turn four of these portions were united to a internal training set. A logistic regression model was trained on the united sets and validated on the remaining part. This gives five estimates of the model's accuracy for each balance. The

step was repeated 30 times resulting in 150 estimates for each balance. The mean of these data was calculated. That model giving the best accuracy was selected. After this step $N-1$ features were left.
Feature reduction steps were performed with $N-1, N-2, \cdots$ features as long as balances could be calculated (which is not possible if no feature is available for the numerator or the denominator).
For each step the selected group of features and the accuracy was written to a text file.
Figure 11 shows the typical development of the mean accuracy during the process of feature reduction. As we select feature with the object to improve the accuracy, in the first couple of steps the model quality increases save of minor fluctuations. Then some local optimal models were reached. Passing through a valley better models are found. With too few features the quality crashes. In blue we marked the best model in this run.

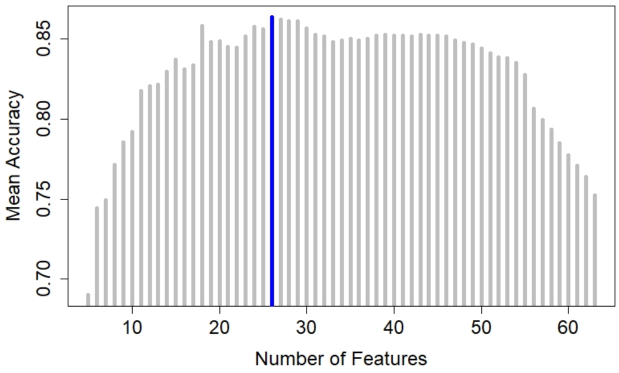

Figure 11: Dependence of Mean Accuracy on feature number. Marked in blue is the best model in this run.

Generally this best model was then further assessed and possibly used in the ensemble learning. In few instances this best model comprised of too many features. Then we looked at the second best model with fewer features (before any assessment).

5.3.4 Validation.

During the process of feature selection some information about model quality was gathered. For each run starting with a certain batch of preselected features the mean accuracy of the best model including n features was written to a text file. The number n runs down form the initial number of preselected features to at least 5. The process stopped early if either the numerator or the denominator categories were empty.

Each sequence of models then contains one model that gave the best results in that run (cf. figure 11). In general this was selected for further validation. If this model contained too many features (more than 30) we took the second best model comprising less features.
These selected models were assessed on the training data as well as on the validation data. For that we calculated the AUROC and a 95% confidence range of AUROC. The ROC curves were plotted in some cases. Mind that due to few instances in the validation data the ROC cures appear to be ragged.
Our first idea was to train the models on the microbiome data only. Later we became interested in the possible influence of several clinical or biometrical information on the predictive power. We then repeated all training runs again this time including the additional data as covariates. For assessing the impact of the covariates we compared best models with and without these additional data. To be precise we optimized the feature selection including the covariates. With these best features at hand we ran the logistic regression on the training data one time including the clinical and biometrical information, one time without that. We think that this is the natural procedure to find the covariates' influence on predictive power.

5.3.5 Results.

Table 7 shows the main results about model assessment. Using the EGGNOG, species and phyla, we got no proper models. In case of phyla this is certainly due to the small amount of features. The information about the microbiome is probably to much condensed and relevant information can not be separated from noise.
The EGGNOG data are at the other end: We guess that information about essentially the same feature is spread over several data fields. Whereas modelling was unsuccessful in case of phyla, using the EGGNOG data we got only poor results. For example including the clinical data, the feature selection resulted in a group of 21 microbiome features. This model gained an AUROC of 0.88 on the training data and of only 0.44 on the validation data. By no means we could find a good model.
Models using the species data were not that bad. Compared with the others shown in the result table, we got a larger gap between AUROCs on training and validation data. As we didn't need them in our ensemble learning model we do not show the results here.
Coming to the results one first observes the usual behaviour: Models perform better on training data than on validation data. Even our models do. More interesting is to see that in case of *Genera* data, there's not much difference between

- the first best model comprising 35 features out of the group of 35 selected features: AUROCs are 0.88 and 0.72 and
- the second best model containing only 18 features of the group of 35 selected features: AUROCs are 0.88 and 0.75.

Data Sets		Number of Features			Preselection Parameters				Assess. Training			Assess. Validation		
feature selection	model	Presel.	Sel.	Model	p-val.	min. out.	max. out.	min $q_{.25}$	AUROC	95% CI		AUROC	95% CI	
Genera & Clinical	Genera & Clinical	65	35	35	0.5	1	5	–	0.88	0.82	0.98	0.72	0.56	0.89
Genera & Clinical	Genera & Clinical	65	35	18	0.5	1	5	–	0.88	0.82	0.94	0.75	0.60	0.91
Genera & Clinical	Genera	65	35	18	0.5	1	5	–	0.76	0.67	0.84	0.61	0.42	0.80
GO	GO	87	17	17	0.1	–	5	-26	0.81	0.73	0.88	0.75	0.56	0.94
KO	KO	66	26	26	0.1	–	6	-21	0.83	0.76	0.91	0.77	0.60	0.93
Clinical	Clinical	–	–	–	–	–	–	–	0.74	0.65	0.82	0.64	0.45	0.84

Table 7: Result table. In *Data Sets* we noted which type of data were used for feature selection and for modelling. Mind that we some times included clinical data to the feature selection but excluded them from modelling. *Number of Features* indicate how many features were used in the distinct steps (preselection, selection and modelling). The *preselection parameters* are listed in the next couple of columns: p-value threshold for sieving, minimal number of outlier, maximal number of outliers and minimal first quartile. The results on assessment of models come next. Here we present the AUROC and its 95% confidence interval boundaries. The assessment was carried out on the training data and the validation data as well.

The AUROC on validation data in this case seems to be better with fewer features. But one should also consult the confidence intervals, and that renders the difference questionable. Nevertheless it would be a nice idea that models with fewer features show less tendency to over-fitting.

5.4 SELBAL.

We owe much to the papers [14] and [13]. In fact we came across with that when we had tried the feature selection process using a multiple logistic regression. These calculations were very slow and the results quite poor. As the *SELBAL* package for *R* provides a fast and convenient approach to results we were encouraged to try our data. And in fact we got some promising results. So we were confident to find reasonable information in our data. Soon we missed the possibility to assess *SELBAL* results using validation data. It was easy to solve that but alas the results were quite poor. Grace to the paper [14] we learned about using balances in regression analysis. After the first calculations using some first preselection rules and our stepwise feature selection in connection with balances we were happy about the promising results and compared some with *SELBAL*. We feel that some more investigations are needed. So we postpone the publication of our results about *SELBAL*.

5.5 Ensemble Learning.

Ensemble Learning is a mean in the area of machine learning, that tries to put several models together to one model. The hope is to get a better model by this merger. Each individual model might have some power on a part of the data and perform badly on an other part. The idea is to find the final model in a way that it takes the best properties from its parts or at least that flaws of the individual models might averaged out.
For a short introduction to the concepts cf. [11]. Our approach might be described as *stacking* (cf. [17]). In fact we found no reference for the method here applied. But the idea is so easy and we claim no priority for that.
Consider the logistic regression models $LRM_m, m = 1, 2, \ldots, M$. For each model and each participant let $\lambda_m(k)$ be the predicted likeliness of participant k to be assigned to the positive outcome group. Then the idea is to put the outcomes $oucome(k)$ as response variables and $\text{logit}(\lambda_m(k))$ for all models as explanatory variables to a logistic model. Here the function

$$\text{logit}(x) = \log \frac{x}{1 - x}$$

is the inverse for the logistic function.
In this manner it is possible to build a new model even if some response variables were used for several individual model. Whether this would be an advisable strategy is an other question.
Like with individual models we trained the ensemble model with training data and assessed the quality using the validation data.

5.5.1 Results.

Comparing the assessments from table 7 with that in 8 it reveals that our ensemble learning approach gives a better model at least when all our models were put together. The ensemble model comprising the microbiome data performs better than any of its parts – at least on the training data. On the validation data there is hardly a difference to *GO*.

Data Sets	Assess. Training		Assess. Validation	
	AUROC	95% CI	AUROC	95% CI
Genera & GO & KO	0.87	0.80 0.93	0.75	0.58 0.92
Genera & GO & KO & Clinical	0.92	0.87 0.97	0.80	0.65 0.94

Table 8: Result table on ensemble learning

If the microbiome data models were merged with the clinical data model, we get the best results. On the validation data the AUROC is 0.80 and the lower boundary of the 95% confidence interval is 0.65. This a promising result.

References

[1] John Aitchison. The statistical analysis of compositional data. *Journal of the Royal Statistical Society. Series B (Methodological)*, 44(2):139–177, 1982.

[2] John Aitchison. *The Statistical Analysis of Compositional Data.* Chapman and Hall, London, 1986.

[3] Annette J. Dobson and Adrain G. Barnett. *An Introduction to Generalized Linear Models..* Chapman & Hall / CRC Press, 2008.

[4] Juan José Egozcue and Vera Pawlowsky-Glahn. Groups of parts and their balances in compositional data analysis. *Mathematical Geology*, 37(7):795–828, Oct 2005.

[5] Peter Filzmoser, Karel Hron, and Matthias Templ. *Applied Compositional Data Analysis.* Springer Nature Switzerland, Cham, 2018.

[6] Gregory B. Gloor, Jean M. Macklaim, Vera Pawlowsky-Glahn, and Juan José Egozcue. Microbiome datasets are compositional: And this is not optional. *Frontiers in Microbiology*, 8:2224, 2017.

[7] Michael Greenacre. *Compositional Data Analysis in Practice.* Chapman and Hall (CRC), Boca Raton., 2019.

[8] Trevor Hastie, Robert Tichiriani, and Jerome Friedman. *The Elements of Statistical Learning. Data Mining, Inference, and Prediction.* Springer, New York, 2001.

[9] Max Kuhn. *caret: Classification and Regression Training*, 2021. R package version 6.0-88.

[10] Vera Pawlowsky-Glahn, Juan José Egozcue, and Raimon Tolosana-Delgado. *Lecture Notes on Compositional Data Analysis.* 2007.

[11] Robi Polikar. Ensemble learning. *Scholarpedia*, 4(1):2776, 2009. revision #186077.

[12] Hefin I. Rhys. *Machine Learning with R, the tidyverse and mlr.* Manning, Shelter Island., 2020.

[13] Javier Rivera-Pinto, M. Luz Calle, and A. Susin. Vignette for selbal package., 2019.

[14] Javier Rivera-Pinto, Juan José Egozcue, Vera Pawlowsky-Glahn, Roger Paredes, Marc Noguera-Julian, and M. Luz Calle. Balances: a new perspective for microbiome analysis. *mSystems*, 3(4), 2018.

[15] John W. Tukey. *Exploratory Data Analysis.* Addison-Wesley, Boston., 1977.

[16] Karl Gerald van den Boogart and Raimon Tolosana-Delgado. *Analysing Compositional Data with R.* Springer, Berlin, 2013.

[17] David H. Wolpert. Stacked generalization. *Neural Networks*, 5(2):241–259, 1992.
